# Editorial: CD24 in the regulation of cellular development and disease

**DOI:** 10.3389/fimmu.2024.1374701

**Published:** 2024-02-27

**Authors:** Sherri L. Christian, Geraldine Cambridge

**Affiliations:** ^1^ Department of Biochemistry, Memorial University of Newfoundland, St. John’s, NL, Canada; ^2^ Department Medicine, University College London, London, United Kingdom

**Keywords:** CD24, ovarian cancer, Siglec, myalgic encephalomyelitis/chronic fatigue syndrome (ME/CFS), T cell priming, immune evasion

CD24 (heat stable antigen or nectadrin) is a GPI-linked, highly glycosylated, dynamically expressed molecule present on cells of the immune system, myeloid cells (including granulocytes), keratinocytes, adipocytes, and also on astrocytes, microglia and neurons (1).

The biological functions of CD24 cover a wide spectrum largely due to the nature and extent of glycosylation. CD24 lacks an intracellular signaling domain, but interestingly shows a superior ability to co-localize with, and organize, a wide range of cell surface molecules into lipid rafts associated with signaling pathways (1). Expression of CD24 is largely differentiation-associated, for example, it is most highly expressed on early lymphocyte progenitors and on neuronal cells during neurogenesis and regeneration (2).

In B cell development, CD24 expression was first used a marker for pre-B cells in fetal liver and bone marrow (3), and subsequently has become one of the main CD markers for staging B cell development (4). CD24 is high on progenitor B cells with slightly lower expression on immature B cells followed by a sharp decease at the naive stage. CD24 is re-expressed but with different functional properties on memory B cells. The signaling properties of CD24 change during B cell development, being linked to pro-apoptotic pathways in pre-B cells but not at later stages (5–7). Moreover, Mensah et al. previously showed a clear increase in CD24 expression in both naïve and memory IgD+B cell populations in the circulation of individuals with myalgic encephalomyelitis/chronic fatigue syndrome (ME/CFS) (8).

In primary bone marrow B cells and B cell lines, CD24 increases the release of extracellular vesicles, which are able to transfer signalling competent receptors to recipient B cells (9–11). In cultured memory B cells, there is a link between CD24 expression and energy metabolism (glucose consumption) and with the metabolic stress response (pAMPK), and possibly senescence, in pre-switch memory B cells (12). CD24 is notably absent from mature plasma cells due to the dependence of niche occupancy on the interaction between CXCR4 (CD184) in lipid rafts on plasma cells and pro-survival signaling via C-X-C chemokine ligand 12 (CXCL12, SDF-1) in the bone marrow (13). CD24 expression excludes CXCR4 from lipid rafts so loss of CD24 is essential to allow access of developing B cells and mature plasma cells to supportive niches.

In the context of malignancy, CD24 expression is widespread, being expressed on approximately 70% of solid tumours and often associated with increased metastatic potential and poor prognosis (14). CD24 is, thus, useful for non-invasive fluorescence imaging, allowing improved tumour resection and as a focus for targeted therapy. When highly expressed on tumour cells, CD24 transmits a ‘do not eat me’ signal via Sigleg-10 receptors on macrophages; an *in trans* interaction between the two cell types (15). Therefore, as well as the possibility of targeting CD24 expressed on tumours using cytotoxic anti-CD24 biologics, the use of ‘blocking’ antibodies to CD24 could also be used as an enhancing agent for immunotherapy (16).

CD24 can also differentiate between the damage associated molecular pattern (DAMP) response through binding to released self-proteins, eg. HMGB-1 and heat shock proteins, but not pathogen associated molecular patterns (PAMPs) (17, 18). Specifically, binding to released HMGB-1 and heat shock proteins allows co-localisation, *in cis*, with Sigleg-10 in dendritic cells. This triggers the downregulation of proinflammatory signalling via the Src homology 2 domain-containing phosphatase 1 (SHP)-Sigleg-10 pathway. The soluble molecule complexed with Fc fragment of IgG (CD24Fc), therefore, can block or blunt a broad inflammatory response leading to CD24Fc being used in several contexts including severe COVID-19 infection and autoinflammatory diseases (19, 20).

Under this Research Topic, there are two review articles and two original research articles. Liu and Zheng's review article focuses on the interactions between CD24 and Siglecs. CD24 is GPI-linked and, therefore, it must interact with a partner *in cis* to mediate signal transduction in the host cell. The authors review the literature demonstrating that CD24 functions via the CD33 family of Siglec proteins in various cell types. In particular, the observation that CD24 functions via Siglec-G/10 to inhibit the dendritic cell response to DAMPs and not PAMPs is highlighted as this paved the way for therapeutic targeting of CD24 via the CD24Fc protein to moderate immune activation. They then discuss the role of CD24 in autoimmune disorders and metabolic function.

In the second review article, Gu et al. discuss the association of CD24 with ovarian cancer. In ovarian cancer, increased CD24 expression is associated with increased metastasis and poorer survival. As the authors discuss, one reason for this observation may be because of the role CD24 plays in immune evasion via the “don’t eat me” signal towards macrophages. The authors finish by discussing other potential roles for CD24 in directly regulating ovarian cancer growth and progression.

In the original research article by Zhang et al. it was demonstrated that expression of CD24 on dendritic cells contributed to priming of T cells. Specifically, they showed that in the absence of CD24, adoptively transferred murine T cells do not undergo sufficient expansion and exhibit increased cell death. This phenotype was rescued when CD24 was re-expressed on dendritic cells.

Lastly, Armstrong et al. found that upon *in vitro* activation with T-dependent or Toll-like-receptor 9 (TLR)-dependent agonists CD24 expression on B cells from ME/CFS patients had a delayed decrease, consistent with *in vivo* results. Additional metabolomic studies further confirmed an association of CD24 with B cell metabolism, namely a correlation with glucose usage and lactate production. B cells from ME/CFS patients also had significantly higher uptake of amino acids, which has been associated with an increased degradation of nucleotides to AMP perhaps showing a possible link with fatigue in patients with ME/CFS. Thus, alterations in CD24 expression may potentially be useful in exploring B cell immunometabolism.

This Research Topic provides a window into the diverse functions of CD24 as well as increasing our knowledge of how CD24 directly regulates immune and innate cell interactions with tumour cells and contributes to T cell priming. Moreover, CD24 may also be linked with immunometabolism, particularly in B cells.

## Author contributions

SC: Writing – original draft, Writing – review & editing. GC: Writing – original draft, Writing – review & editing.
